# Flash glucose monitoring system: impact on glycemic control and body mass index in type 1 diabetes mellitus

**DOI:** 10.20945/2359-3997000000405

**Published:** 2021-09-29

**Authors:** Juliana Marques Sá, Sara Campos Lopes, Mariana Barbosa, Inês Ferreira Barros, Maria Joana Santos

**Affiliations:** 1 Departamento de Endocrinologia do Hospital de Braga Braga Portugal Departamento de Endocrinologia do Hospital de Braga, Braga, Portugal

**Keywords:** Diabetes, type 1 diabetes, flash glucose monitoring, glycated hemoglobin, body mass index

## Abstract

**Objective:**

Flash glucose monitoring (FGM) is increasingly used in type 1 diabetes mellitus (T1D) management. This study aimed to assess glycated hemoglobin (HbA1c) and body mass index (BMI) in the first year of FGM use in patients with T1D and to identify predictive factors of benefit associated with its use.

**Subjects and methods:**

Retrospective study of T1D patients, using FGM for ≥ 6 months and under intensive insulin therapy with multiple daily injections.

**Results:**

In 179 patients with a median (Md) age of 43.0 years (P25 31.0; P75 52.0) and disease duration of 18.0 years (P25 10.0; P75 28.0), initial HbA_1_c was 7.9% (P25 7.2; P75 8.8) and initial BMI was 24.0 kg/m^2^ (P25 21.9; P75 26.2). With FGM, HbA1c improved significantly to 7.6% (P25 7.0; P75 8.3) at 6 months and 7.7% (P25 6.95; P75 8.5) at 12 months (p < 0.05), with more patients with HbA_1_c < 7% (16.1% vs 22.5%) and fewer patients with HbA_1_c ≥ 8% (49.1% vs 35.8%) (p < 0.05). Initial HbA_1_c 8.0-8.9% (HR 1.886; 95% CI 1.321-2.450) and ≥ 9.0% (HR 3.108, 95% CI 2.454-3.761) predicted greater HbA_1_c reduction. BMI increased significantly, especially between 6 and 12 months (BMI Md 23.8 [P25 21.9; P75 26.2] kg/m^2^ and 24.0 [P25 22.0; P75 26.2] kg/m^2^, respectively) (p < 0.05). Overweight (HR 4.319, 95% CI 3.185-5.453) and obesity (HR 8.112, 95% CI 3.919-12.306) predicted greater weight gain.

**Conclusions:**

FGM use was associated with significant improvement in HbA_1_c, mainly in patients with worse previous glycemic control. It was also associated with increased BMI, especially if baseline BMI ≥ 25 kg/m^2^, so weight control strategies should be emphasized.

## INTRODUCTION

Self-monitoring of blood glucose (SMBG) has long been one of the key elements of type 1 diabetes mellitus (T1D) management. This method enables patients to assess their blood glucose levels at any time using finger-stick blood samples, test strips and glucose meters ( 1 , 2 ). In recent years, however, the appearance of new technologies, such as flash glucose monitoring (FGM), which facilitate the monitoring of interstitial glucose, has changed the lives of many patients with T1D ( 2 , 3 ). Whereas SMBG provides isolated blood glucose values, FGM, through painless scanning, provides considerably more information, such as the direction and velocity of glycemic changes (i.e., trend arrows), estimated glycated hemoglobin (HbA_1_c), average glucose, percentage of readings above, below and within the predefined target range, number and duration of hypoglycemic events and daily profiles ( 2 ). This allows for a reduction in hypoglycemic events and glucose variability, as well as an improvement in glycemic control, quality of life and treatment satisfaction ( 2 , 4 , 5 ).

The most modern FGM devices demonstrate a mean absolute relative difference in glucose values of 11.4% compared to SMBG, allowing the collected data to be used for self and hetero adjustment of insulin doses ( 6 ). Changes in interstitial glucose are seen with a delay of approximately 5 minutes relative to blood glucose, so there are situations in which there is a higher and lower correlation between the values provided by FGM and SMBG, namely in the first 24 hours of use, extreme values (hypoglycemia and hyperglycemia) and when rapid changes in glucose levels occur. SMBG measurements are recommended to confirm the values obtained via FGM when interstitial glucose readings change rapidly, to confirm hypoglycemia and when symptoms do not correspond to FGM values ( 2 , 5 , 7 ).

Scanning the FGM device several times a day was associated with better HbA_1_c values, higher percentage of time in the glycemic target range, less time in nocturnal hypoglycemia and less severe hypoglycemic events (<55 mg/dL) ( 5 , 8 – 10 ). Moreover, recent studies have shown that FGM use is associated with a substantial reduction in HbA_1_c, particularly in individuals with higher HbA_1_c values before its use ( 5 , 11 ).

Currently, our endocrinology department follows about 600 patients diagnosed with T1D. Most of our patients have access to FGM devices, which have been reimbursed by the government in Portugal since January 2018.

The main aim of this study was to assess the impact of continuous FGM device use for at least 6 months in T1D patients and to identify predictive factors of benefit associated with the use of this technology.

## SUBJECTS AND METHODS

### Study design and participants

This observational and retrospective study’s target population included patients with T1D who underwent follow-up at the Endocrinology Department of Hospital de Braga, Portugal.

Study eligibility criteria were T1D diagnosis, being 18 years or older, using a FGM device continuously for at least 6 months and undergoing intensive insulin therapy by multiple daily injections (MDI). Exclusion criteria included intermittent or less than 6 months of device use, being an insulin infusion pump carrier, pregnancy, initiation or suspension of hypoglycemic drugs (e.g., metformin, glucagon-like peptide-1 [GLP-1] analogs or sodium-glucose co-transporter 2 inhibitors [iSGLT-2]) and/or change in the basal insulin type during the analyzed period.

All patients were treated with a basal-bolus regimen (long-acting and rapid-acting insulin analogues), and we subdivided the patients into 2 groups: “functional insulin therapy” and “fixed doses”. “Functional insulin therapy” refers to a regimen in which patients know how to count carbohydrates and use the concepts of carbohydrate-to-insulin ratio and insulin sensitivity factor, which could be different for each meal ( 12 ). The term “fixed doses” refers to treatment used for patients who did not count carbohydrates and who used a fixed table to know how much rapid-acting insulin they should take, according to glycemic level and specific meal.

The data were collected by consulting electronic clinical records. The study was performed according to a protocol properly approved by the local ethics committee (reference 226_2019).

### Outcomes

Our primary outcome was the longitudinal evolution of HbA_1_c at 6 and 12 months after initiation of FGM use. Our secondary outcome was the longitudinal evolution of BMI, as a surrogate marker of weight. Possible predictive factors for the greatest benefit of the use of this technology, namely sex, functional insulin therapy, age, diabetes duration, initial HbA_1_c, initial BMI, variation of long-acting insulin dose and variation of BMI or HbA_1_c, were also evaluated for each outcome.

### Statistical analysis

Collected data was analyzed using IBM SPSS^®^ version 26.0 (IBM Corp., Armonk, NY, USA) and STATA IC^®^14 software (StataCorp, College Station, Texas, USA).

For continuous quantitative variables, we assessed the presence of normal distribution through histogram analysis, the Shapiro-Wilk test and asymmetry and kurtosis evaluations. Because the data were distributed non-normally, we present our results using the median, 25^th^ and 75^th^ percentiles, as well as minimum and maximum values. For categorical variables, we present absolute numbers and percentages. We divided patients into subgroups according to age, disease duration and HbA_1_c value considering the 25th, 50th and 75th quartiles of the distribution of our sample in these variables. For BMI, we used the cutoffs recommended by the World Health Organization ( 13 ).

For comparisons between groups, we used Fisher’s exact test for categorical variables and the Kruskal-Wallis test for continuous variables. To compare paired samples at various follow-up times, we used the Wilcoxon test.

Models of univariate longitudinal regression adjusted for the initial value of HbA_1_c, and BMI were constructed. Multivariate longitudinal regression models were also constructed to analyze possible predictors of the variation of HbA_1_c and BMI after FGM device use.

We used a 95% confidence interval and considered a result statistically significant if p < 0.05.

## RESULTS

The population’s baseline characteristics are presented in Table 1 . We included 179 patients with a median (Md) age of 43.0 years and a median duration of disease of 18.0 years. Approximately 33.5% of the patients were under a functional insulin therapy regimen. The initial median HbA_1_c was 7.9% (P25 7.2; P75 8.8), and 49.1% of patients presented initial HbA_1_c ≥ 8%. The initial median BMI was 24.0 kg/m^2^ (P25 21.9; P75 26.2), and 39% of patients were overweight or obese. All our patients used FGM at least for 6 months, and 74.3% of them used it for 12 months or more.

**Table 1:** Patient characteristics at baseline

Sex		
	Male	94 (52.5%)
	Female	85 (47.5%)
Age (years) a		43 (31; 52) (18;80)
Age (groups)		
	≤29	40 (22.4%)
	30-39	33 (18.4%)
	40-49	52 (29.1%)
	≥50	54 (30.2%)
Diabetes duration (years) a		18 (10; 28) (1; 62)
Diabetes duration (groups) (years)		
	≤9	41 (22.9%)
	10-19	55 (30.7%)
	20-29	41 (22.9%)
	≥ 30	42 (23.5%)
Treatment with functional insulin therapy		60 (33.5%)
Long-acting insulin		
	Glargine	153 (85.5%)
	Detemir	21 (11.7%)
	NPH	5 (2.8%)
Long-acting insulin daily dose (U) a		22 (16; 30) (6; 68)
Long-acting insulin daily dose (U/kg/day) a		0.32 (0.26; 0.45) (0.11; 0.90)
HbA_1_c (%) a		7.9 (7.2; 8.8) (5.6; 13.8)
HbA_1_c (groups) (%)		
	<7.0	26 (16.2%)
7.0-7.9	56 (34.8%)	
	8.0-8.9	42 (26.1%)
	≥9.0	37 (23.0%)
Weight (kg) a		67.3 (59.3; 74.5) (48.4; 97.8)
BMI (kg/m^2^) a		24 (21.9; 26.2) (17.2; 33.7)
BMI (Groups) (kg/m^2^)		
	<25.0	97 (61.1%)
	25.0-29.9	54 (34.0%)
	≥30.0	8 (5.0%)
FGM usage time (months)		
	6-11	46 (25.7%)
	12-17	62 (34.6%)
	18-23	54 (30.2%)
	≥24	17 (9.5%)

a Median (P25, P50) (min; max).

To analyze the HbA_1_c and weight variation, we assessed the variation of the long-acting insulin dose, since this variable affects both HbA_1_c and weight. The daily long-acting insulin dose (U/kg/day) did not undergo statistically significant changes over the 12 months analyzed (p = 0.111 from 0 to 6 months and p = 0.078 from 6 to 12 months). The doses of rapid-acting insulin analogues were not analyzed statistically, as some patients were treated with functional insulin therapy, while others used insulin in fixed doses.

### Evolution and predictors of change in HbA1c with FGM use

**Figure 1:**
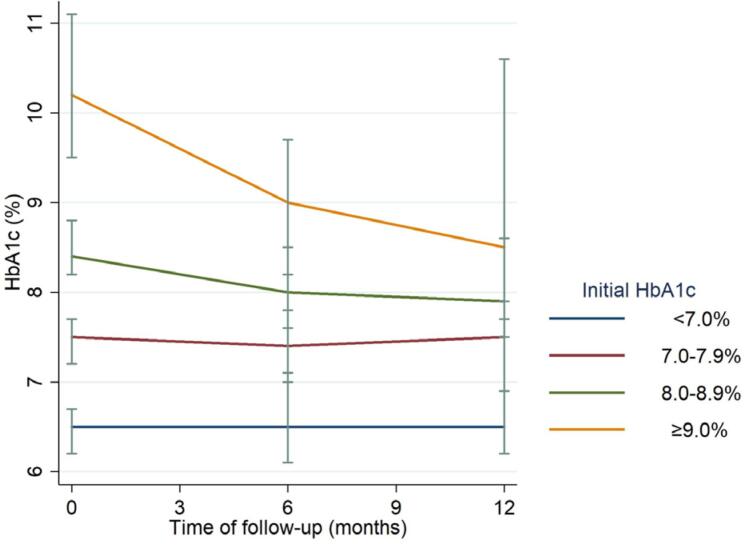
Longitudinal evolution of HbA1c during the 12 months of follow-up.

During the first year of FGM use, HbA_1_c decreased significantly in our population, especially in the first 6 months of use ( Table 2 ; Figure 1 ). We observed a significant increase in the number of patients with HbA_1_c < 7% (16.1% at baseline vs. 22.5% at 6 months, p < 0.001) and a reduction in the number of patients with HbA_1_c ≥ 8% (49.1% at baseline vs. 37.1% at 6 months, p < 0.001). From 6 to 12 months of analysis, the trend remained, but it lacked statistical significance.

**Table 2:** Longitudinal evolution of HbA_1_c by subgroup (univariate longitudinal regression adjusted to the initial HbA1c value)

HbA_1_c		Initial	6 months	12 months	HR	CI	p
Global		7,9 (7,2; 8,8) (5,6; 13,8)	7,6 (7,0; 8,3) (5,7; 16,4)	7,7 (6,95; 8,5) (5,9; 13,7)	-0.127	-0.237; -0.016	<0.05
Sex							
	Male	7,7 (7,1; 8,6) *	7,6 (6,9; 8,2)	7,5 (6,9; 8,4)	0.0136	-0.201; 0.228	0.901
	Female	8,3 (7,4; 9,3) *	7,6 (7,2; 8,6)	7,8 (7,2; 8,6)			
Treatment with functional insulin therapy							
	No	7,9 (7,3; 9,3)	7,6 (7,1; 8,3)	7,8 (7,2; 8,5)	-0.022	-0.241; 0.197	0.844
	Yes	7,9 (7,1; 8,7)	7,5 (7,0; 8,1)	7,6 (6,8; 8,5)			
Age (groups)							
	≤29 years	8,0 (7,2; 9,3)	7,4 (7,0; 8,5)	7,6 (6,8; 8,5)	-0.018	-0.113; 0.077	0.710
	30 – 39 years	8,2 (7,2; 9,3)	7,6 (7,0; 8,6)	7,6 (7,2; 8,5)			
	40 – 49 years	8, 0 (7,4; 8,7)	7,7 (6,95; 8,3)	7,8 (6,9; 8,6)			
	≥50 years	7,6 (7,1; 8,6)	7,6 (7,3; 7,9)	7,9 (7,4; 8,4)			
Diabetes duration (groups)							
	≤9 years	8,0 (7,3; 9,5)	7,8 (7,2; 8,8)	7,6 (7,2; 8,5)	-0.094	-0.187; -0.001	p < 0.05^a^
	10-19 years	8,0 (7,5; 8,8)	7,8 (7,3; 8,3)	7,7 (7,2; 8,3)			
	20-29 years	8,2 (7,2; 9,0)	7,6 (6,8; 8,2)	8,2 (6,8; 8,7)			
	≥30 years	7,6 (6,9; 8,2)	7,5 (6,8; 7,9)	7,5 (6,7; 7,9)			
BMI (groups)							
	<25.0 kg/m^2^	8,1 (7,5; 9,3)	7,9 (7,3; 8,5) *	7,9 (7,3; 8,6) **	-0.014	-0.177; 0.204	0.889
	25.0-29.9 kg/m^2^	7,7 (7,2; 8,6)	7,5 (6,8; 7,8) *	7,4 (6,9; 7,9) **			
	≥30.0 kg/m^2^	8,1 (7,4; 9,5)	8,2 (7,9; 9,7) *	8,8 (7,6; 9,0) **			
Initial HbA_1_c (groups)							
	<7%	6,5 (6,2; 6,7) *	6,5 (6,1; 7,1) *	6,5 (6,2; 6,9) *	1.012	0.889; 1.144	<0.001^b^
	7-7.9%	7,5 (7,2; 7,7) *	7,4 (7,0; 7,8) *	7,5 (6,9; 7,9) *			
	8-8,9%	8,4 (8,2; 8,8) *	8,0 (7,6; 8,5) *	7,9 (7,5; 8,6) *			
	≥9%	10,2 (9,5; 11,1) *	9,0 (8,2; 9,7) *	8,5 (7,7; 10,6) *			

Median (p25; p75) (min; max).

* p < 0.05.

** 0.05 < p < 0.1.

a p < 0.05 between 20-29 years.

b p < 0.001 between 8-8.9% and ≥ 9%.

We constructed multiple univariate longitudinal regression models adjusted to initial HbA_1_c for each potential predictor of its evolution ( Table 2 ). Gender, modality of treatment and initial BMI were not associated with a favorable HbA_1_c evolution. Patients who had T1D for 20-29 years had worse HbA_1_c evolution (p < 0.05). Patients with the highest HbA_1_c at baseline (8-8.9% and ≥ 9%) had remarkably positive HbA_1_c evolution (p <0.001) ( Table 2 ).

A multivariate longitudinal regression model for possible confounding factors for the absolute variation of HbA_1_c in the first 12 months of FGM use ( Table 3 ) confirmed that patients with HbA_1_c 8.0-8.9%, especially those with HbA_1_c ≥ 9%, showed the greatest improvement in glycemic control. Factors such as initial BMI of 25-29.9 kg/m^2^ (overweight) and an increase in BMI in the first 6 months (BMI positive variation) had a negative effect on the evolution of HbA_1_c ( Table 3 ).

**Table 3:** Multivariate longitudinal regression model for possible confounding factors of the evolution of HbA1c during the 12 months of follow-up

Factor		Coefficient	CI	p
HbA1c evolution 0-6 months		-0.475	-0.762; -0.187	0.001
HbA1c evolution 6-12 months		-0.337	-0.629; -0.045	0.024
Initial HbA1c (%) a				
	7.0-7.9	1.124	0.578; 1.670	<0.001
	8.0-8.9	1.886	1.321; 2.450	<0.001
	≥9.0	3.108	2.454; 3.761	<0.001
Sex		0.042	-0.353; 0.437	0.836
Treatment with functional insulin therapy		-0.162	-0.543; 0.269	0.462
Age (years)		-0.012	-0.030: 0.005	0.171
Diabetes duration (years)		-0.005	-0.026; 0.015	0.612
Initial BMI (kg/m^2^) b				
	25-29.9	-0.397	-0.793; -0.001	0.049
	≥30.0	-0.657	-1.850; 0.536	0.281
BMI variation 0-6 months		-0.374	-0.598; -0.149	0.001
BMI variation 6-12 months		-0.087	-0.350; 0.176	0.517
Dose variation of long-acting insulin 0-6 months(U/kg/d)		2.098	-1.102; 5.298	0.199
Dose variation of long-acting insulin 6-12 months (U/kg/d)		-3.749	-8.312; 0.815	0.107

a Relative to initial HbA1c group < 7.0%.

b Relative to initial BMI < 25 kg/m^2^.

### Evolution and predictors of change in BMI following FGM use

During the first 12 months of FGM use, there was a significant increase in the absolute value of BMI, especially between 6 and 12 months ( Table 4 ). Although it lacked statistical significance, there was also a decrease in the number of patients with normal weight (61% at baseline vs. 57.3% at 12 months) and an increase in the number of overweight or obese patients (39% at baseline vs. 42.8% at 12 months) (p > 0.05).

**Table 4:** Longitudinal evolution of BMI by subgroups (univariate longitudinal regression adjusted to the initial BMI value)

BMI		Initial	6 months	12 months	HR	CI	p	
Global		24,0 (21,9; 26,2) (17,2; 33,7)	23,8 (21,9; 26,2) (16,5; 33,3)	24,0 (22,0; 26,2) (17,1; 33,1)	0.124	0.0245; 0.223		<0.05
Sex								
	Male	24,0 (22,1; 25,7)	23,7 (21,8; 26,1)	24,3 (22,2; 25,9)	0.0413	-0.163; 0.246		0.692
	Female	24,1 (21,8; 26,7)	23,8 (22,1; 26,6)	23,8 (21,8; 26,9)				
Treatment with functional insulin therapy								
	No	24,1 (22,4; 26,4)	23,8 (22,1; 26,6)	24,3 (22,3; 26,5)	-0.032	-0.252; 0.188		0.776
	Yes	23,9 (20,5; 25,9)	23,8 (21,1; 25,8)	23,8 (20,5; 25,7)				
Age (groups)								
	≤29 years	23,8 (21,7; 24,5) **	23,6 (21,8; 24,9)	23,2 (21,9; 25,4) *	-0.0604	-0.156; 0.035		0.213
	30–39 years	22,9 (20,9; 25,4) **	23,6 (21,7; 25,9)	22,2 (21,3; 25,0) *				
	40–49 years	25,2 (22,6; 27,4) **	24,4 (22,3; 27,8)	25,3 (22,5; 28,2) *				
	≥50 years	24,2 (22,6; 26,2) **	23,8 (22,2; 26,0)	25,0 (23,3; 26,3) *				
Diabetes duration (groups)								
	≤9 years	23,8 (22,1; 25,4) *	23,4 (22,1; 25,4) **	23,8 (22,1; 25,5)	-0.012	-0.1097; 0.086		0.814
	10-19 years	23,5 (21,6; 25,4) *	22,4 (21,7; 25,4) **	22,8 (21,7; 25,8)				
	20-29 years	23,9 (22,4; 27,0) *	24,0 (22,4; 26,6) **	24,9 (21,5; 27,0)				
	≥30 years	25,5 (23,2; 27,5) *	25,0 (23,0; 27,2) **	25,1 (23,6; 27,2)				
Initial BMI (groups)								
	<25 kg/m^2^	22,6 (20,9; 23,8) *	22,6 (21,2; 23,8) *	22,5 (21,1; 23,9) *	4.484	3.997; 4.969		<0.05 a
	25-29.9 kg/m^2^	26,4 (25,6; 27,6) *	27,0 (25,6; 28,4) *	26,5 (25,3; 28,5) *				
	≥30 kg/m^2^	31,5 (30,3; 32,7) *	31,2 (30,6; 31,6) *	31,4 (31,2; 31,8) *				
Initial HbA1c (groups)								
	<7%	24,2 (22,3; 25,2)	23,0 (22,0; 24,9)	23,6 (21,6; 25,1)	0.122	0.0165; 0.227		<0.05 b
	7-7.9%	25,1 (22,1; 27,4)	24,0 (22,0; 28,1)	24,1 (22,4; 27,3)				
	8-8,9%	24,0 (21,5; 26,4)	24,2 (22,1; 27,8)	24,6 (23,0; 26,1)				
	≥9%	23,9 (21,6; 25,2)	23,4 (21,4; 25,6)	23,5 (22,2; 26,2)				

* p < 0.05.

** 0.05 < p < 0.1.

a Group 25-29.9 kg/m^2^ and ≥ 30 kg/m^2^.

b Group 8-8.9% and ≥ 9%.

We constructed multiple univariate longitudinal regression models for each potential predictor of BMI evolution, adjusting for initial BMI. The longitudinal evolution of the BMI had no relation to gender, modality of treatment, patient’s age or disease duration. Patients with overweight or obesity and HbA_1_c ≥ 8% at baseline showed a significant increase in BMI over the first 12 months of FGM use ( Table 4 ).

A multivariate longitudinal regression model for possible confounding factors of BMI variation showed that patients with BMI ≥ 25 kg/m^2^ at baseline gained more weight over the follow-up period ( Table 5 ).

**Table 5:** Multivariate longitudinal regression model for possible confounding factors of the evolution of BMI during the 12 months of follow-up

Factor		Coefficient	CI	p
BMI evolution 0-6 months		0.257	0.025; 0.489	0.030
BMI evolution 6-12 months		0.373	0.141; 0.605	0.002
Initial HbA1c (%) a				
	7.0-7.9	1.053	-0.603; 2.709	0.213
	8.0-8.9	0.986	-0.644; 2.616	0.236
	≥9.0	0.988	-1.048; 3.024	0.342
Sex		0.181	-0.987; 1.350	0.761
	Treatment with functional insulin therapy	-0.905	-2.206; 0.397	0.173
	Age (years)	-0.013	-0.062; 0.036	0.598
	Diabetes duration (years)	-0.008	-0.064; 0.049	0.791
Initial BMI (kg/m^2^) b				
	25-29.9	4.319	3.185; 5.453	0.000
	≥30.0	8.112	3.919; 12.306	0.000
HbA1c variation 0-6 months		-0.365	-0.861; 0.131	0.149
HbA1c variation 6-12 months		0.188	-0.483; 0.859	0.583
Dose variation of long-acting insulin 0-6 months (U/kg/d)		2.578	-5.764; 10.920	0.545
Dose variation of long-acting insulin 6-12 months (U/kg/d)		11.173	-2.964; 25.310	0.121

a Relative to initial HbA1c group < 7.0%.

b Relative to initial BMI < 25 kg/m^2^.

The doses of rapid-acting insulin analogues were analyzed in patients with fixed doses who gained weight and in patients who maintained or lost weight. At baseline, rapid-acting insulin dose per kg was 0.22 U/kg (P25 0.14; P75 0.40) in patients who gained weight and 0.23 U/kg (P25 0.13; P75 0.40) in those who maintained or lost weight (p ≥ 0.05). The variation of rapid-acting insulin doses per kg in the first 12 months in patients who gained weight was also not statistically different from those who maintained or lost weight (Md -0.002 U/kg [P25 -0.03; P75 0.06] vs Md 0.01 U/kg [P25 0.00; P75 0.03], respectively, p ≥ 0.05).

## DISCUSSION

The use of continuous glucose monitoring devices is associated with improvements in glycemic control in patients with T1D treated with MDI ( 10 , 14 ). In line with previous publications ( 5 , 11 ), our study demonstrated a statistically significant and clinically relevant improvement in HbA_1_c after one year of FGM use, especially in individuals with the worst initial metabolic control (HbA_1_c ≥ 8%). In fact, initial HbA_1_c ≥ 8% was a predictor of greater benefit, regardless of patients’ other characteristics, whereas overweight or weight gain in the first 6 months negatively affected the evolution of HbA_1_c. Through FGM technology, this group of patients gained access to additional and useful glucose data with a more convenient and painless method compared to SMBG, possibly giving them more motivation to increase the number of glucose assessments and more confidence to auto-adjust short-acting insulin doses. A possible increase in short-acting insulin doses could explain the HbA_1_c improvement seen over the 12 months of follow-up because all types of insulin are associated with weight gain ( 15 , 16 ). These adjustments may have been made by the patients or by their physicians, who also had access to more information with FGM than with SMBG. Because the short-acting insulin doses were not evaluated in all patients (it was not possible to know the exact dose of rapid-acting insulin in patients on functional insulin therapy), we did not analyze that factor, so this is merely a hypothesis.

In fact, previous data demonstrated that FGM use resulted in changes in the timing of bolus administration of rapid-acting insulin, which was associated with better HbA_1_c values ( 17 , 18 ). The fact that our patients were using this technology for the first time, with access to new information such as trend arrows and a graph with glycemic values throughout the day, might also explain the HbA_1_c improvement, especially in the first 6 months, when the device was completely novel, and more readings were probably taken per day. Obese patients consistently presented worse HbA_1_c, which is likely associated with greater food intake, a more sedentary lifestyle and greater insulin resistance.

Regarding weight, there was an increase in BMI over the follow-up time, especially in months 6-12. The only predictive factor for weight gain after starting FGM was BMI ≥ 25 kg/m^2^ at baseline. Although, to our knowledge, no studies have specifically evaluated the longitudinal evolution of weight after the start of FGM, a publication aimed at evaluating the impact of FGM in people with T1D in real-life conditions also found a slight increase in BMI at 6 months of follow-up ( 5 ). This weight increase was not caused by an increase in rapid-acting insulin doses, at least in patients on fixed-dose regimens, but it could be associated with the fear of hypoglycemia, which would justify greater food intake and less physical activity ( 16 ). Patients with poor glycemic control (HbA_1_c ≥ 8%) and overweight or obese patients experienced statistically significant weight increases throughout the period analyzed.

The strengths of this study included its considerable sample size, long follow-up time and the analysis of longitudinal variation of BMI beyond the evolution of HbA_1_c, which had not been studied so carefully before. However, there are also some limitations, such as the absence of a control group, no evaluation of rapid-acting insulin analogue doses in all patients, the study’s retrospective design and the impossibility of guaranteeing that HbA1c and weight evaluations were performed using the same method and equipment. Furthermore, we have no information regarding lifestyle changes (e.g., diet, exercise) that may have contributed to the findings of our study. The fact that it was a retrospective study also limited the collection of information regarding the glycemic variability, time in range and number of hypoglycemic events. Although we cannot know exactly, most patients used the FGM at least 5 times daily (before breakfast, lunch, afternoon snack, dinner and bedtime) but we have no data that allow us to establish relationships between the number of scans and the results obtained in our work. It would also be interesting to analyze the educational status of our sample in a future study and check if there are differences in glycemic control according to an academic degree.

In summary, in our study of the first year of FGM use, we found a significant improvement in HbA1c that was consistent over the 12-month period but more evident in the first 6 months. Patients with previous worse glycemic control experienced major improvements, whereas patients with initial overweight and those who increased their weight during the first 6 months experienced fewer benefits with this technology. FGM use was also associated with weight gain, especially in patients who were overweight or obese at baseline. Therefore, when starting FGM, weight control strategies such as a physical exercise plan and dietary changes should be considered in this specific group of individuals.
